# *Escherichia coli* RelA Regulation *via* Its C-Terminal Domain

**DOI:** 10.3389/fmicb.2020.572419

**Published:** 2020-11-03

**Authors:** Ilana Kaspy, Gad Glaser

**Affiliations:** Department of Developmental Biology and Cancer Research, Institute for Medical Research Israel-Canada, Hebrew University of Jerusalem, Jerusalem, Israel

**Keywords:** RelA-C-terminus domain, stringent response, *Escherichia coli*, *relA*, (p)ppGpp

## Abstract

One of the most important stress responses in bacteria is the stringent response. The main player in this response is the signal molecule (p)ppGpp, which is synthesized by a Rel family protein. In *Escherichia coli*, RelA is the main synthetase of (p)ppGpp in response to amino acid starvation. Although the synthetic activity of RelA is well-understood, its regulation is not yet fully characterized. The C-terminus domain (CTD) of the *E. coli* RelA is responsible for the regulation of the protein and for its complete dependency on wild-type (WT) ribosome. The CTD contains three Cysteine residues, positioned in a very conserved order. Together with our previous results, we show *in vitro* the negative dominant effect of a part of the WT CTD (AA 564–744) named YG4 on RelA synthetic activity. This effect is abolished using mutated YG4 (YG4-638). *In vitro* and mass spectrometry (MS)-MS analysis of the native RelA and the mutated RelA in Cys-638 (Rel638) in the presence of the native and mutated YG4 (YG4-638) reveals that RelA forms a homodimer *via* its CTD by the formation of a disulfide bond between the two Cys-638 residues. This supports our previous data which showed, using a two-hybrid system, interactions between RelA proteins *via* the CTD. Finally, we show *in vitro* that excess of the native YG4 inhibited RelA synthetic activity but did not affect the amount of RelA bound to the ribosome. Our results suggest that the regulatory mechanism of RelA is by the dimerization of the protein *via* disulfide bonds in the CTD. Upon amino-acid starvation, the dimer changes its conformation, thus activating the stringent response in the cell.

## Introduction

To survive, bacteria must be able to respond to changes in their environment. Depriving *Escherichia coli* of one or more amino acids (AAs) triggers the stringent response (Stent and Brenner, 1961; Cashel, 1969; Cashel and Gallant, 1969; Kaspy et al., 2013). Within a few seconds after the onset of amino-acid starvation, one can observe the accumulation of phosphorylated derivatives of GTP and GDP, collectively called (p)ppGpp (Cashel, 1969; Cashel and Gallant, 1969; Fiil et al., 1972; Lund and Kjeldgaard, 1972). The transcription factor DksA and (p)ppGpp bind together to RNA polymerase (RNAP; Metzger et al., 1988; Gentry et al., 1993; Gourse et al., 1996; Paul et al., 2004) affecting a large number of physiological activities, most particularly transcription (Pedersen and Kjeldgaard, 1977; Gentry et al., 1993; Magnusson et al., 2005). (p)ppGpp is important not only in overcoming nutritional deprivation but has a role also in virulence, survival during host infection, antibiotic resistance, and formation of persister cells (Dalebroux et al., 2010; Dalebroux and Swanson, 2012; Kaspy et al., 2013).

In *E. coli* and other proteobacteria, (p)ppGpp synthesis is driven by RelA, a 84 kDa ribosome-associated enzyme (Alfoldi et al., 1962; Metzger et al., 1988). RelA is activated in response to amino-acid starvation (Cashel and Gallant, 1969; Fiil et al., 1972; Lipmann and Sy, 1976). Uncharged tRNAs bind to the ribosomal “A” site, stalling protein synthesis (Haseltine et al., 1972; Haseltine and Block, 1973) and stimulating a reaction in which, within seconds, RelA synthesizes (p)ppGpp (Fiil et al., 1972). In extracts of normally growing cells, RelA is associated with a small fraction (about 1%) of the ribosomes (Pedersen and Kjeldgaard, 1977). Both physically and functionally, *E.coli* RelA includes two distinct domains: the N-terminal domain [NTD; amino acids (AAs) 1-455], which is responsible for (p)ppGpp synthesis and the C-terminal domain (CTD; AAs 405–744), which is responsible for regulating RelA activity (Metzger et al., 1989; Schreiber et al., 1991; Gropp et al., 2001). When RelA bears a mutation in amino acid Gly-251, it lacks synthetic activity both *in vivo* and *in vitro* (Wendrich and Marahiel, 1997; Gropp et al., 2001). The open reading frame (ORF) of RelA is known to end with an amber codon which, when suppressed, yields a longer protein containing 771 AA that is no longer regulated (Metzger et al., 1988). Although the stringent response has been investigated for over 50 years, the regulatory mechanism of RelA responsible for the synthesis of the key regulator of this response is still not fully understood. Much work has been devoted to trying to decipher the regulatory mechanism of the Rel protein family (Wendrich et al., 2002; Agirrezabala et al., 2013; Turnbull et al., 2019; Takada et al., 2020).

**Figure 1:**
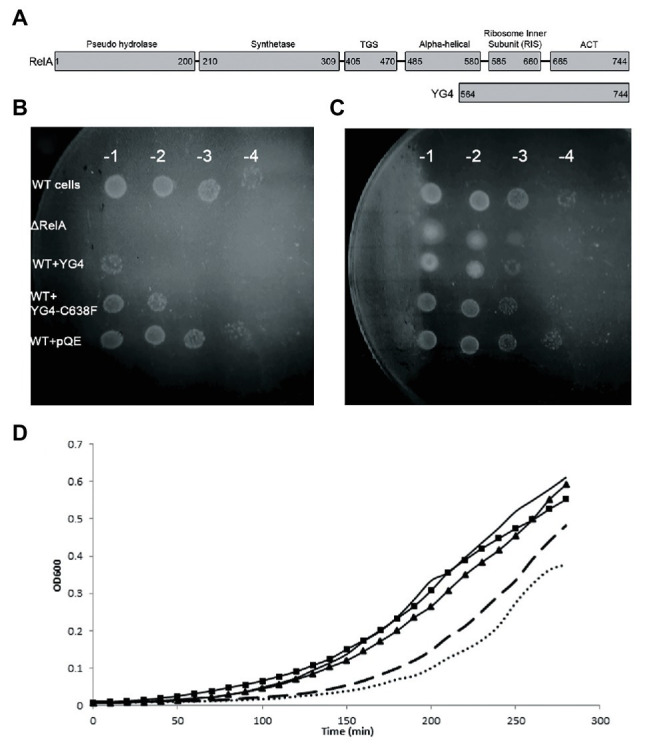
Over expression of RelA-C-terminus domain (CTD) on cell growth. **(A)** RelA domains as described in Loveland et al. (2016) and YG4 fragment. **(B,C)** W3110 [wild type (WT)] cells bearing a plasmid overexpressing YG4 or YG4-C638F were grown in Luria-Bertani (LB) medium for 2 h, after which overexpression was induced by the addition of 1 mg/ml of IPTG for 1 h. Cells were collected and washed, diluted in serial dilutions, and plated on M9 medium. ΔRelA and WT + pQE (an empty plasmid) were used as controls. **(B)** containing AT; **(C)** without AT. All plates were incubated at 37°C overnight. WT cells with an “empty” plasmid (WT) and cells deleted for RelA (ΔRelA) were used as controls. **(D)** All cell types were grown in duplicates in a 24-well plate in LB medium supplemented with 100 μg/ml of ampicillin. After 2 h of growth, all cells were supplemented with 1 mg/ml of IPTG and were grown for an additional 2.5 h at 37°C with shaking. Cell growth was monitored by optical density (OD) measuring OD_600_. No bullets – WT cells with an empty plasmid; square (▪) – WT cells overexpressing YG4; triangle (▲) – WT cells overexpressing Rel251; dashed – WT cells overexpressing Rel-C638F; dots – WT cells overexpressing RelA.

Here, we shed more light on the regulatory mechanism of *E. coli* RelA. The CTD of *E. coli* RelA can be divided into four domains that were shown to interact with ribosome at different sites, and are responsible for RelA binding to the ribosome (Agirrezabala et al., 2013; Arenz et al., 2016; Brown et al., 2016; Loveland et al., 2016). It was shown previously that overexpression of the RelA CTD in wild-type (WT) cells starved for AAs causes a reduction in the accumulation of (p)ppGpp (Gropp et al., 2001). A mutation in the conserved sequence AA 612–638, in which Cys-638 is replaced by phenylalanine (RelA-C638F) leads to the constitutive ribosome-independent synthesis of (p)ppGpp. Thus, the RelA CTD cannot regulate the production of (p)ppGpp without AA Cys-638 (Gropp et al., 2001). Moreover, in earlier bacterial two-hybrid system experiments, we found that a fragment of the CTD, YG4 (AA 564–744, MW 21 kDa; Figure 1A), is involved in RelA-RelA interactions (Gropp et al., 2001). The YG4 fragment, which inhibited RelA synthetic activity, contains two of the four domains; the ribosome inter-subunit (RIS; AA 585–660) and the ACT domain (AA 665–744), as described previously (Loveland et al., 2016). Both of these domains bind near the A and P sites of the ribosome. According to Cryo-EM data, Cys-638 is part of an α-helix structure in the RIS domain that docks into the A-site finger. The other two domains (TGS and AH AA 405–580) are also found inside the ribosome and connect the YG4 part to the synthetase domain (Loveland et al., 2016; Figure 1A). The NTD does not form a clear structure under normal translation, but upon binding of an uncharged tRNA to the A-site, RelA undergoes conformational change, stabilizing the NTD in order for it to synthesize (p)ppGpp (Agirrezabala et al., 2013; Loveland et al., 2016). Recent work suggests that RelA is incapable of self-oligomerization and that the regulatory mechanism is likely in *cis* by intramolecular interactions, rather than in *trans* (Turnbull et al., 2019). In that report, the authors used the “full length” CTD (containing all four domains; Turnbull et al., 2019). In contrast, in our and other’s previous results, no interaction was observed between the NTD and the full length RelA or between the NTD and the YG4 (Gropp et al., 2001; Yang and Ishiguro, 2001; Jain et al., 2006). Together with our present *in vitro* study, we show that YG4 inhibits (p)ppGpp synthesis without competing for ribosome binding of the full length RelA. Furthermore, we found that Cysteine residues in the CTD, especially Cys-638, are essential for RelA regulation and the formation of disulfide bonds between CTDs.

## Materials and Methods

### Strains and Plasmids

As we have described previously (Gross et al., 2006), all of our vectors contain a 6-his tag coding sequence between the start codon and the multi-linker for the desired gene cloning. Cloning the proteins, especially RelA, after the addition of a His tag does not affect the regulation of the proteins (Schreiber et al., 1991).

### Media

Luria-Bertani (LB), LB-agar (from BIO101) or M9 minimal media were used for growth media. When required, these media were supplemented with either 100 μg/ml ampicillin or 50 μg/ml kanamycin. To induce nutritional stress in liquid culture, 1 mM serine hydroxamate (SHX; Tosa and Pizer, 1971) was added. Selection for resistance to 3-amino-1,2,4-triazole (AT) was performed on minimal M9 AT plates containing 15 mM AT and all amino acids except histidine, as described previously (Gross et al., 2006).

### Growth Curves

W3110 or CF9467 cells bearing different plasmids as indicated in the results section were grown in LB medium at 37°C with shaking (Table 1). The optical density was measured using TECAN device every 10 min. At OD_600_ of 0.2, 1 mg/ml of isopropyl-β-D-1-thiogalactoside (IPTG) was added to induce the overexpression of the proteins, and growth was monitored for the indicated time period. For the colony forming assay, 1 h after protein induction by IPTG, cells were collected and washed in saline three times, diluted in serial dilutions, and plated on M9-agar plates in the presence or absence of 3-amino-1,2,4-Triazole (AT). All plates were incubated at 37°C overnight, and colony formation was monitored.

**Table 1:** Bacterial strains.

Strain name	Genotype	Source
W3110	WT *lacI*^q^::Kan^R^	Laboratory collection
CF9467	W3110∆*relAlacI*^q^::Kan^R^	Schreiber et al., 1991

### Protein Purification

*Escherichia coli* CF9467 cells were transformed with pQE_30_-*relA*, pYG4, or pYG4-C638F (Table 2) and were grown to mid-exponential phase at 37°C with shaking in LB medium supplemented with 100 μg/ml ampicillin. The expression of his-tagged RelA, YG4, or YG4-C638F was induced by the addition of 1 mM IPTG. After 2 h of growth at 37°C, the cells were harvested by centrifugation, resuspended in buffer A (20 mM Naphosphate buffer pH 7.4, 0.5 M NaCl, 10 mM imidazole), supplemented with protease inhibitor cocktail Complete EDTA free (Roche Diagnostics), and then sonicated. To remove cell debris and unbroken cells, lysates were centrifuged at 10,000 *g* for 15 min. Supernatants were loaded onto Ni-NTA agarose columns (Qiagen). The columns were washed with buffer A containing 20 mM imidazole, and his-tagged protein was eluted with 250 mM imidazole in buffer A. The protein-containing fractions were analyzed by SDS page, and then pooled and dialyzed against buffer B (100 mM Tris-HCl pH 8.5, 10 mM EDTA, 1 mM DTT and 25% glycerol). Final protein concentrations were measured using the Bio-Rad Protein Assay dye reagent.

**Table 2:** Plasmids.

Plasmid name	Relevant characteristics	Source
pQE_30_	::amp	Qiagen
pYG4	pQE_30_ carrying His-tagged YG4 under tac promoter::amp	Gropp et al., 2001
pYG4-C638F	pQE_30_ carrying His-tagged YG4-C638F under tac promoter::amp	Gropp et al., 2001
pRelA	pQE_30_ carrying His-tagged RelA under tac promoter::amp	Gropp et al., 2001
pRelA-C638F	pQE_30_ carrying His-tagged RelA-C638F under tac promoter::amp	Gropp et al., 2001
pRel251	pQE_30_ carrying His-tagged RelA-G251E under tac promoter::amp	Gropp et al., 2001

### Lowsalt Crude Ribosome Preparation

Crude ribosomes are ribosomes associated with both mRNA and tRNA. These were prepared as described by Block and Haseltine (1975) with the following modifications: ∆*rel*A cells were grown in LB medium with shaking at 37°C. At OD_600_ = 1.5, the cell culture was centrifuged at 4,000 *g* at 4°C for 20 min and frozen overnight at −70°C. The pellet was resuspended in cold buffer R [consisting of 100 mM Tris-acetate pH 8, 10 mM Mg(AcO)_2_ and 1 mM DTT]. Lysozyme, supplemented with protease inhibitor cocktail Complete EDTA free (Roche Diagnostics), was added to a final concentration of 3 mg/ml, and cells were sonicated. Cell lysates were centrifuged at 12,000 *g* for 40 min to remove cell debris and unbroken cells. The supernatants were centrifuged in a Beckman Ti-65 rotor at 28,000 g at 4°C for 4 h. The pellets were resuspended in buffer R and were incubated at 4°C overnight. To remove excess of membrane residues, all of the suspended pellets were combined together and centrifuged at 8,000 *g* at 4°C for 15 min. The supernatant from this centrifugation was then centrifuged again, using a sucrose cushion, at 4°C for 4 h in a Beckman Ti-65 rotor at 30,000 g. The final pellet, containing the purified ribosomes was then resuspended in buffer R, and the ribosomal concentration was determined based on RNA measurements in an ND-1000 Spectrophotometer (Nano-Drop). The ribosomes were frozen and stored at −70°C.

### *In vitro* RelA Activity Assay

For the *in vitro* RelA activity assay, reaction buffer (RM) was used containing 0.5 mM GTP, 4 mM ATP, 50 mM Tris-HCl (pH 7.4), 1 mM DTT, 10 mM MgCl_2_, 10 mM KCl, and 27 mM (NH_4_)_2_SO_4_. For each reaction, 10 μCi of (α-^32^P)GTP was added. In a total volume of 20 μl, 1 μg of purified RelA or purified RelA-C638F was mixed together with RM, 30 μg of ribosomes and varying amounts of YG4, YG4-C638F, or RelA-G251E proteins. After 1 h of incubation at room temperature, the reactions were stopped by the addition of 5 μl of formic acid reaching a final concentration of 20%. 5 μl aliquots of each reaction were loaded and separated on Cellulose PEI TLC plates (Merck) using 1.5 M KH_2_PO_4_ as mobile phase. The plates were autoradiographed using the Fijix Bas100 PhosphorImager (Japan); the (p)ppGpp content was determined based on relative intensities calculated using TINA 2.0 software (Raytest).

### Ribosome Binding Assay

*In vitro* reactions containing increasing concentrations of either YG4 or YG4-C638F were carried out as described above for the RelA activity assay but without the addition of radio-labeled GTP. The reaction mixtures were centrifuged at 30,000 *g* at 4°C for 4 h. The soluble fractions were removed, and ribosomal samples from the pellets were separated by 12% SDS-polyacrylamide gel electrophoresis, transferred to PVDF membrane (Millipore), and processed for immunoreaction using mouse-anti-His monoclonal antibody (GE Healthcare). Immuno-reactive proteins were detected using a chemi-luminescence kit (Biological Industries) according to the protocol of the manufacturer.

### *In vitro* Cross-Linking

Protein cross-linking was carried out in a 10 μl reaction mixture containing 12.5 mM Naphosphate pH 7.2, 12.5 mM NaCl, 2.5% glycerol, 1 × 10^−3^% glutaraldehyde, and 4 μg of protein. After 15 min of incubation in ice, each sample was loaded onto SDS polyacrylamide gel and electrophoresed for further Western Blot Analysis, as described above.

### Mass Spectrometric Analysis (MS-MS)

A sample of YG4 dimers after cross-linking with glutaraldehyde as mentioned earlier was divided into two. To cleave possible disulfide bonds, dithiothreitol (DTT) was added to one of the samples; the second sample was left untreated. Both samples were digested with Trypsin. The peptide mixtures were solid phase extracted using C18 resin filled tips (ZipTip Milipore) and subsequently nanosprayed into the Orbi-trap MS system in 50% acetonitrile containing 1% formic acid.

Mass spectrometry (MS) was carried out with Orbi-trap (Thermo Finnigen) using a nanospray attachment. Data analysis was done using bioworks 3.3 package, and database searches were performed against the NCBInr database with Mascot package (Matrix Science).

## Results

### Dominant Negative Effect of RelA-CTD on the Stringent Response

Our previous data showed a dominant negative effect of RelA-CTD fragment (YG4) on RelA activity in *E. coli* (Gropp et al., 2001). Following this, we overexpressed YG4 and YG4-C638F in WT *E. coli* cells and plated them on M9 medium together with 3-amino-1,2,4-Triazole (AT; Figure 1B), thereby creating histidine starvation conditions. It was clear that under these conditions, cells overexpressing YG4 exhibited difficulties in overcoming the AA starvation (by three orders of magnitude) unlike cells overexpressing YG4-C638F (by two orders of magnitude; Figure 1B). Under the same conditions without AT a less negative effect on cell growth was observed (one order of magnitude in presence of YG4 or YG4-C638F; Figure 1C). This phenomenon can have two possible explanations: (i) YG4 binds to RelA, thus inhibiting its activity on the ribosome and (ii) YG4 competes with RelA for ribosome binding. Both theories are valid for explaining poor RelA activity. Additionally, in order to overexpress the proteins, all genes were cloned under a *lac* promoter, and IPTG was used to induce their overexpression. Massive overexpression following the use of a *lac* promoter and IPTG can interrupt normal cell activity, regardless of the target itself and cause different effects on cell function. In order to rule out this theory, we plated the same cells on M9 medium without AT. Cells overexpressing YG4 or YG4-C638F showed the same growth rate (Figure 1C), indicating that the overexpression itself probably did not affect the cell growth, although a slight growth arrest was seen on M9 medium without AT (Figure 1C) We next examined the effect of overexpression of alternate Rel proteins on *E. coli* growth in rich medium. As previously shown (Schreiber et al., 1991), overexpression of an active full-length RelA, such as the WT RelA (Figure 1D dots) or Rel-C638F (Figure 1D dashed) displayed delayed growth as compared to the WT cells that showed no overexpression at all (Figure 1 solid line, no bullets). This can be explained by the production of (p)ppGpp in those cells, which is known to inhibit cell growth. However, when RelA bearing a mutation in position 251(Gropp et al., 2001; Figure 1D triangles) that renders the protein incapable of synthetic activity, or YG4 was overexpressed (Figure 1D squares), no effect on growth rate was observed. Meaning that an excess of a protein lacking synthetic activity, in this case, did not inhibit cell growth in rich medium. The lac-IPTG system is known to produce large amounts of proteins in bacterial cells which, in some cases, can inhibit cell growth, especially in poor medium, such as M9. Our results indicate that while overexpression itself of these proteins does not affect cell growth, cell growth is inhibited by (p)ppGpp synthesis.

### Cys-638 in *E. coli*-RelA-CTD Is Essential for Protein Regulation

**Figure 2:**
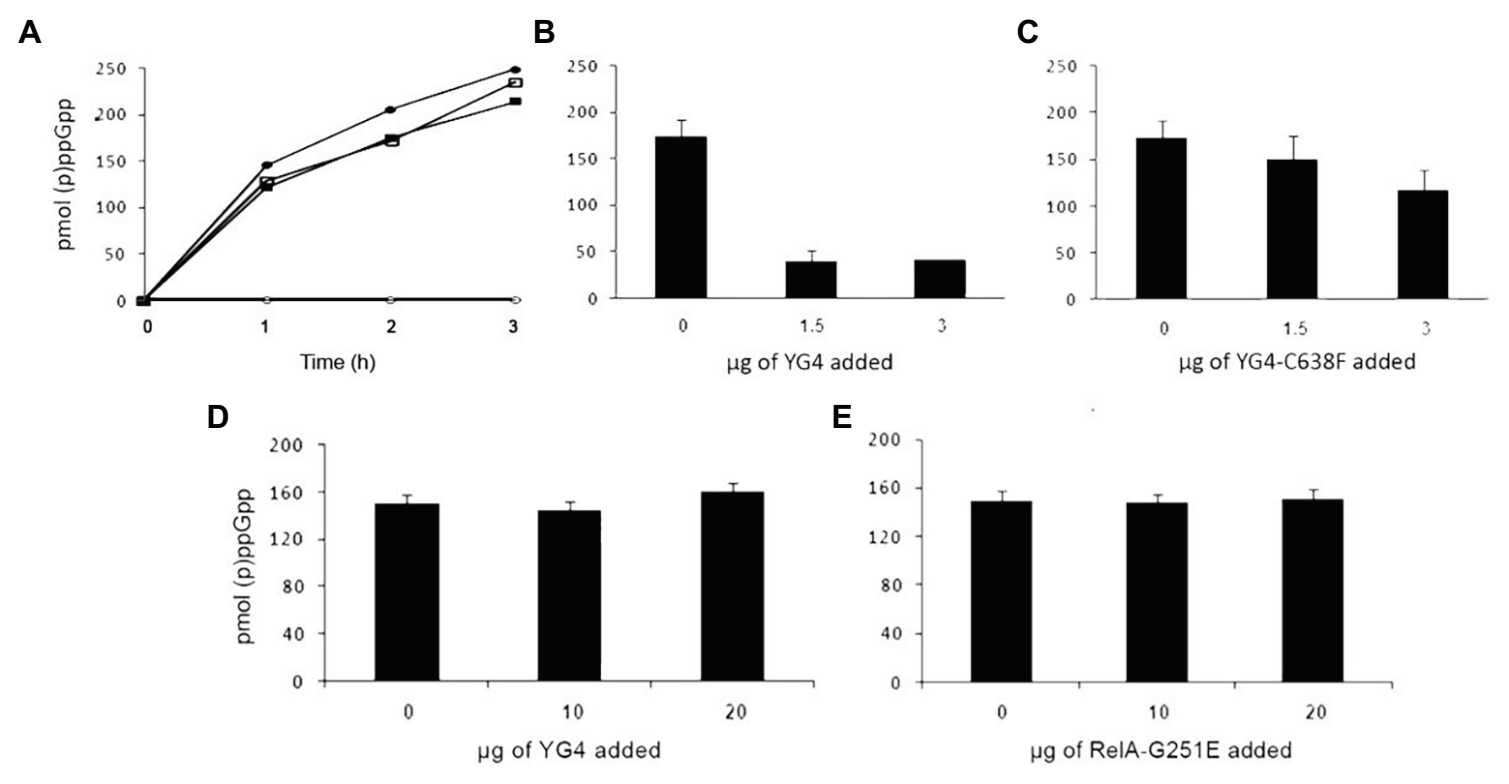
Dominant negative effect of CTD on RelA synthetic activity *in vitro*. (p)ppGpp production by RelA *in vitro*. **(A)** Solid circles (●) WT-RelA in the presence of ribosomes; empty circles (o) – WT-RelA in the absence of ribosomes; solid squares (▪) – Rel-C638F in the presence of ribosomes; empty squares (▫) – Rel-C638F in the absence of ribosomes. **(B,C)** (p)ppGpp production by 1 μg of RelA with the addition of increasing amounts of **(B)** YG4 and **(C)** YG4-C638F. **(D,E)** (p)ppGpp production by 1 μg of Rel-C638F with the addition of increasing amounts of **(D)** YG4 and **(E)** Rel251.

We next examined what the effect of an excess of RelA variants or fragments was on *E. coli* RelA activity *in vitro*. In order to synthesize (p)ppGpp, the *E. coli* RelA must be activated by a stalled ribosome. Protein binding to the ribosome is *via* its CTD (Wendrich and Marahiel, 1997) and as described more recently *via* the RIS and the ACT domain (Loveland et al., 2016). Three Cys residues are present in the CTD and extremely conserved throughout the Rel protein family (Atkinson et al., 2011). The importance of all three Cys residues in the CTD was shown in previous publications (Gropp et al., 2001; Atkinson et al., 2011), but the strongest effect on RelA activity and the RelA-RelA interaction was observed by a single mutation in Cys-638 (Gropp et al., 2001). Thus, in the present study, we chose to focus on the C638F mutation. We first tested the synthetic activity of both WT RelA and Rel-C638F *in vitro*, focusing on the regulatory effect of the YG4, especially on the role of Cys-638. When examining the synthetic activity of the mutated Rel-C638F *in vitro*, the protein lacked regulatory activity, producing (p)ppGpp in a ribosome-independent manner as compared to the WT RelA (Figure 2A; Gropp et al., 2001). The replacement of a single amino acid was sufficient in rendering the protein ribosome-independent, showing that Cys-638 is essential for the regulation of RelA activity. We next examined the synthetic activity of both proteins following the addition of YG4. The results correlated with our previous data showing that, where (p)ppGpp production by RelA in the presence of YG4 (Figure 2B) was poor, there was almost no effect on its synthetic activity in the presence of YG4-C638F (Figure 2C). The synthetic activity of Rel-C638F was not affected by either the presence of YG4 or Rel251 that supplies WT CTD (Figures 2D,E), which may be a hint to the lack of ability of Rel-C638F to form RelA-RelA interactions. In all cases, the additional protein was in a greater excess (at least 1:6 molar ratio) than the synthetase in the reaction. These results indicate that a change in Cys-638 causes the reversal of the protein YG4’s dominant negative effect on the synthetic ability of a ribosome-dependent protein.

### Excess of CTD During (p)ppGpp Production Does Not Affect the Amount of RelA on the Ribosomes

**Figure 3:**
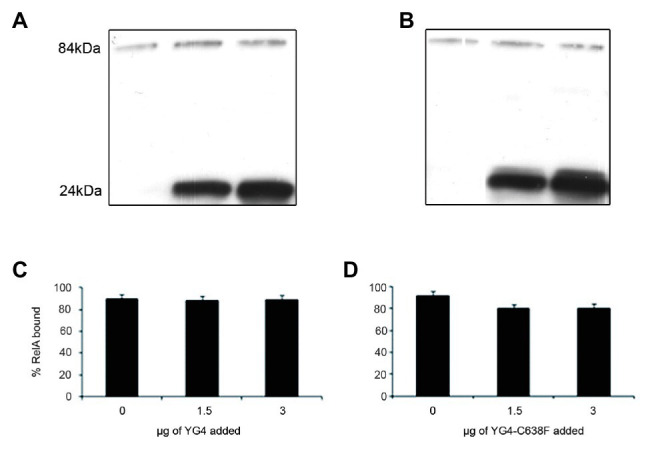
RelA binding to the ribosomes. Western blot analysis of the ribosomal fraction from an *in vitro* reaction of (p)ppGpp synthesis by 1 μg of RelA, with the addition of increasing amounts of **(A)**. YG4 and **(B)** YG4-C638F as indicated in **(C)** and **(D)**, respectively. **(C,D)** Quantification of RelA bound to the ribosome from **(A)** and **(B)**, respectively.

When performing *in vitro* (or *in vivo* in previous publications; Gropp et al., 2001) activity tests, we usually employ a substantial excess of the YG4. This could possibly explain the inhibition of RelA activity as being the result of this excess YG4 competing with RelA for ribosomal binding. This contradicts the theory that this inhibition is the result of the YG4 forming an “incorrect” dimer with RelA. In order to test these two theories, we performed a ribosome binding assay with RelA in the presence of the native or the mutated YG4. The reaction included all components of an *in vitro* activity assay. After 45 min of incubation, the ribosomal fraction was separated by centrifugation, and the amount of RelA in each sample was tested by Western blot analysis (Figure 3). Interestingly, neither the excess of the native (Figure 3C) nor the mutated YG4 (Figure 3D) affected RelA’s ability to bind to the ribosome. Another interesting observation is that most of the YG4 or YG4-C638F that was present in the reaction tube was also bound to the ribosomes (Figures 3A,B). These results stand together with the results of RelA activity test in the presence of YG4 and YG4-C638F (Figure 2), thus indicating that the inhibitory effect of YG4 on RelA is by its binding to the protein itself. These results also emphasize YG4’s ability to bind to the ribosome.

### The CTD Forms Dimers *in vitro*

**Figure 4:**
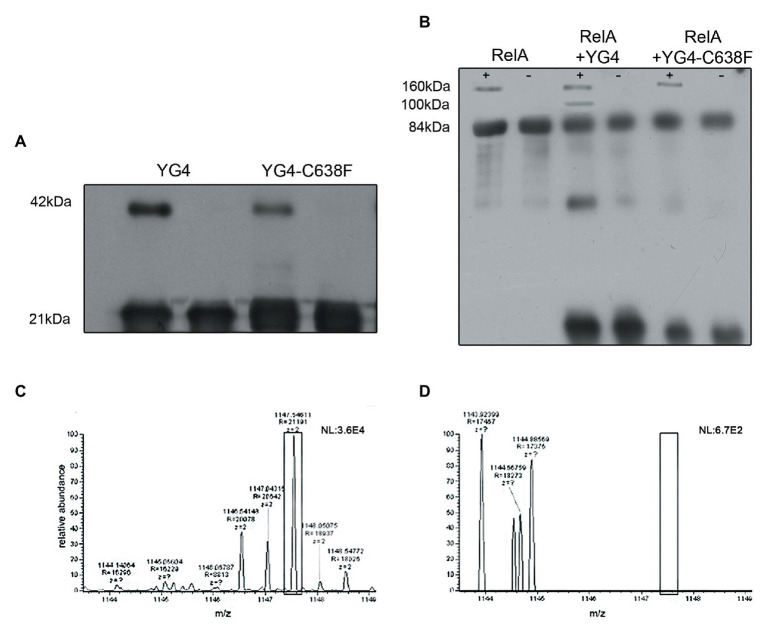
Dimerization of YG4 and RelA. *In vitro* cross-linking reactions were performed by incubating the proteins and analyzing them by Western Blot. (+): with glutaraldehyde; (−): without glutaraldehyde. **(A)** Cross-linking of YG4 (left) and YG4-C638F (right). **(B)** From left to right: cross-linking of RelA, RelA and YG4, RelA, and YG4-C638F. **(C,D)** Mass spectrometry (MS)-MS analysis of digested YG4 peptides. **(C)** MS-MS analysis of YG4 dimers, digested without DTT reducing treatment, showing a fragment including an inter-chain S-S bond corresponding to Cys638-Cys638 (box); m/z = 1,147; **(D)** MS-MS analysis of reduced and digested YG4 dimers; note the absence of the fragment seen in (**C**; see box).

We further wanted to explore whether RelA and YG4 are capable of forming dimers *in vitro*. Cross-linking experiments revealed the formation of dimers for both these proteins (Figures 4A,B). It can be seen that YG4-C638F forms fewer dimers than the native YG4 (Figure 4A). Full-length RelA was also capable of forming homo-dimers, and also hetero-dimers with YG4, which was seen at 100 kDa. This is probably due to the fact that both proteins have a Cys residue at position 638 (Figure 4B). But no dimers with YG4-C638F were observed due to the lack of a Cys residue at position 638 in the mutant YG4 (Figure 4B). These results reinforce the importance of Cys638 for RelA-RelA interactions. The fact that YG4-C638F forms homodimers and that no RelA-YG4-C638F dimers were seen indicates that only WT-YG4 is capable of inhibiting RelA activity, similar to the results of RelA synthetic activity (Figure 2). This probably happens due to the formation of an incorrect dimer between RelA and YG4. It should be noted that we used glutaraldehyde when performing the cross-linking, which is an unspecific cross-linker that covalently links molecules that are present close enough to each other. In both cross-linking experiments (Figures 4A,B), only a small portion, out of the large amounts of protein that were used, formed dimers. It should be noted that the amplified amounts of protein that were used in these experiments are not proportional to the actual protein concentrations in the cell. Our main purpose was to examine the ability of these proteins to interact with each other *in vitro*, based on our previous results (Gropp et al., 2001) and to further investigate the basis for the dimer formation. Thus, we were able to examine whether the dimers were formed specifically due to S-S bonds between two Cys-638. Employing MS-MS analysis, we examined a YG4 dimer that showed the existence of a di-sulfide bond at C-638 only when YG4 dimer was not treated with DTT, which breaks S-S bonds (Figure 4C). But when YG4 dimer was treated with DTT no S-S bond was found (Figure 4D). Based on these findings, it seems that the interactions between YG4-C638F with itself or other proteins are not specific and not strong enough to inhibit RelA synthetic activity.

## Discussion

The stringent response is most likely one of the most important stress responses in bacteria, and, as such, is persistently studied. Due to the fast, synthetic activity of RelA in response to a lack of amino acids, the cell is able to respond very quickly by entering cell-growth arrest, indispensable for its survival. While small amounts of RelA are present in the cell throughout its entire lifetime, it is mostly in a low activity mode. Binding of an uncharged tRNA to the ribosome activates RelA and enables its catalytic activity. Although the stringent response has been studied for over 5 decades, the regulatory mechanism of RelA is poorly understood. The CTD domain is responsible for the regulation and ribosome binding, and is composed of four sub-domains (Atkinson et al., 2011; Loveland et al., 2016). Our previous results (Gropp et al., 2001) showed the importance of the last two domains (AA 564–744), and the importance of the three Cys residues present in the RIS domain (Loveland et al., 2016; Figure 1A), especially in protein-protein interactions. This was also reinforced with recent reports about the involvement of the CTD in the oligomerization of Rel protein in *Mycobacterium* (Singal et al., 2017) and also in the regulation of *Bacillus Subtilis* Rel synthetic activity (Pausch et al., 2020). Here, we closely examined RelA-RelA *via* its CTD interactions *in vitro* by using purified ribosomes (70S) where lack of charged tRNA in the tube mimic amino acid stress conditions. Our results show that *in vitro*, excess of YG4 inhibits synthetic activity of RelA under stress conditions. On the other hand, YG4 did not inhibit cell growth under normal growth conditions *in vivo*, showing that while the excess of protein itself does not affect cell growth, it has a direct effect on RelA synthetic activity. When Cys-638 was replaced by Phenylalanine, this effect was abolished. Moreover, cross-linking experiments and MS-MS analysis revealed the ability of the native RelA and YG4 to form dimers *via* the formation of S-S bonds between Cys-638 both between the full length proteins and between YG4 fragments (Figure 4), which we believe also exist *in vivo*. These observations suggest that a direct interaction between YG4 and RelA causes inhibition of RelA synthetic activity. Finally, a ribosome binding assay showed that the amount of RelA on the ribosome did not change in spite of increasing amounts of YG4 in the reaction tube. These results indicate that the inhibitory effect of the YG4 on RelA is not *via* competitive binding, but rather a direct interaction between RelA and YG4. Taking together our present and previous results, we believe that the regulation of *E. coli* RelA activity is controlled by its CTD, especially by the RIS and ACT domain, which are part of YG4. In all Cryo-EM studies (Agirrezabala et al., 2013; Arenz et al., 2016; Brown et al., 2016; Loveland et al., 2016), RelA was found as a monomer on the ribosome. Together with our results, it appears that the CTD is responsible not only for ribosomal binding of the protein, but also for the oligomerization of the protein, which prevents RelA synthetic activity in the cytosol. Under stress conditions and a binding of an uncharged tRNA to the ribosome, RelA is stabilized, thus enabling its ability to synthesize (p)ppGpp (Loveland et al., 2016). This was also recently shown in Rel protein from *B. subtilis*, where Rel is in an oligomeric state in the cytosol during normal growth conditions, but upon accumulation of uncharged tRNA the dimer dissociates by interaction with the CTD and together binds to a cognate ribosome (Pausch et al., 2020). Here, based on all of our present data together with our previous results (Gropp et al., 2001) and recent studies (Loveland et al., 2016; Pausch et al., 2020), we present a partial model for RelA regulation, which uncovers additional part in the complex “RelA regulation puzzle”. It is likely that RelA forms the dimer only in the cytosol *via* the formation of a disulfide bond with Cys-638 residues. Based on Pausch study (Pausch et al., 2020), dimer is probably separated when RelA-CTD binds an uncharged tRNA in the cytosol, which enables the dissociation of the dimer to monomer which then binds to the ribosome. The dominant negative effect of YG4 on RelA synthetic activity is probably by inhibiting this interaction with an uncharged tRNA, thus interrupting the dissociation of the dimer. The importance of Cys-638 is probably not only in the formation of the disulfide bonds but also in stabilizing RelA structure in order for it to be activated, as exhibited by the ability of Rel-C638F to synthesize (p)ppGpp also in absence of ribosomes (Figure 2A). This is probably due to the fact that Rel-C638F folds in the cytosol as the native RelA does, when bound to a stalled ribosome. A possible explanation of our results in which YG4 inhibits RelA synthetic activity could be due to the fact that the addition of a native YG4 *in vivo* or *in vitro*, creates an “incorrect” dimer which either disables RelA to form a monomer, or disables the conformational change of RelA allowing its synthetic activity on the ribosome. It is possible that the full length CTD is unable to form such interactions with the full length RelA resulting in its inability to inhibit RelA synthetic activity. Taken together all results, it seems that parts in the CTD are responsible for RelA-RelA interactions, which are responsible and important for RelA regulation.

## Data Availability Statement

The raw data supporting the conclusions of this article will be made available by the authors, without undue reservation.

## Author Contributions

GG and IK conceived the study. IK performed all experiments. Both the authors contributed to the article and approved the submitted version.

### Conflict of Interest

The authors declare that the research was conducted in the absence of any commercial or financial relationships that could be construed as a potential conflict of interest.
